# Brain choline concentrations may not be altered in euthymic bipolar disorder patients chronically treated with either lithium or sodium valproate

**DOI:** 10.1186/1475-2832-3-13

**Published:** 2004-07-30

**Authors:** Ren H Wu, Tina O'Donnell, Michele Ulrich, Sheila J Asghar, Christopher C Hanstock, Peter H Silverstone

**Affiliations:** 1 Department of Biomedical Engineering, University of Alberta, Edmonton, Alberta, Canada; 2 Department of Psychiatry, University of Alberta, Edmonton, Alberta, Canada

**Keywords:** Bipolar disorder, lithium, sodium valproate, magnetic resonance spectroscopy, choline

## Abstract

**Background:**

It has been suggested that lithium increases choline concentrations, although previous human studies examining this possibility using ^1^H magnetic resonance spectroscopy (^1^H MRS) have had mixed results: some found increases while most found no differences.

**Methods:**

The present study utilized ^1^H MRS, in a 3 T scanner to examine the effects of both lithium and sodium valproate upon choline concentrations in treated euthymic bipolar patients utilizing two different methodologies. In the first part of the study healthy controls (n = 18) were compared with euthymic Bipolar Disorder patients (Type I and Type II) who were taking either lithium (n = 14) or sodium valproate (n = 11), and temporal lobe choline/creatine (Cho/Cr) ratios were determined. In the second part we examined a separate group of euthymic Bipolar Disorder Type I patients taking sodium valproate (n = 9) and compared these to controls (n = 11). Here we measured the absolute concentrations of choline in both temporal and frontal lobes.

**Results:**

The results from the first part of the study showed that bipolar patients chronically treated with both lithium and sodium valproate had significantly reduced temporal lobe Cho/Cr ratios. In contrast, in the second part of the study, there were no effects of sodium valproate on either absolute choline concentrations or on Cho/Cr ratios in either temporal or frontal lobes.

**Conclusions:**

These findings suggest that measuring Cho/Cr ratios may not accurately reflect brain choline concentrations. In addition, the results do not support previous suggestions that either lithium or valproate increases choline concentrations in bipolar patients.

## Background

Bipolar disorder is a chronic severe mental illness affecting approximately 1% of the adult population. The most widely used mood stabilizer for this condition is lithium [1], although the exact mechanism by which it is clinically effective remains undetermined. One suggestion is that it acts via effects on choline metabolism. This is based upon findings that lithium can inhibit the membrane transport of choline in both animals [2], and human post-mortem brain tissue [3]. It also increases the accumulation of erythrocyte choline in lithium-treated patients [4-7]. Also of note is that choline concentrations increase significantly in rats following electroconvulsive shock [8]. Based upon this data choline has been used to treat mania in a some small pilot studies [9], with one open label study reporting that choline augmentation of lithium treatment helped rapid-cyclers [10]. Patients treated with choline also had increased basal ganglia concentrations of choline, suggesting that externally administered choline could alter brain concentrations [11,12].

The most appropriate method to measure brain choline concentrations *in vivo *utilizes proton magnetic resonance spectroscopy (^1^H-MRS). Previous studies of bipolar patients utilizing this methodology have had mixed findings. Overall, while some studies have suggested there may be increased choline concentrations in specific situations [13-18], more have found no changes [19-27], and one found a trend towards a decrease in concentrations [28]. In both patients and volunteers lithium also doesn't appear to alter choline/creatine peak ratios concentrations [29,30]. Nonetheless, two reviews concluded that the evidence to date suggests that lithium increases brain choline concentrations [31,32], although as noted in these reviews previous studies have varied considerably in terms of patient populations, brain region studied, medications administered, and MRS methodology. Many studies have also examined differing patients (Type I and Type II) in differing mood states (mixed, depressed, manic, and euthymic). This may partially explain the varied results.

Sodium valproate is also widely used as a mood stabilizer, both alone and in combination with lithium [33]. To date there have been few studies which have examined the effects of sodium valproate on choline concentrations or activity. An *in-vitro *study suggested that valproate may inhibit choline acetyltransferase activity [34]. In one study 9 patients taking either lithium or valproate were examined [35], and increased Cho/Cr ratios were seen in the bipolar patients compared to controls. There were no differences between the lithium and valproate treatment groups, although the sample sizes were small. However, another study in epilepsy patients treated with valproate found no changes in choline concentrations [36]. Nonetheless, given the lack of studies to date, the possibility that valproate and lithium may both increase choline concentrations warrants further investigation.

Most of the previous studies have examined Cho/Cr ratios. However, it should be noted that the "choline" resonance peak seen in ^1^H-MRS spectra is composed primarily of phosphocholine and glycerophosphocholine, along with free choline, acetylcholine, and cytidine diphosphate choline. Also, we have shown in animal studies that both lithium and valproate can both decrease creatine concentrations [37]. Therefore, when using Cho/Cr ratios it is not possible to be certain that any changes in this peak represent changes in brain choline concentrations. We were therefore interested to determine if there were any differences in results when using different methodologies, and more specifically to determine if studies using a ratio methodology may have different results from studies utilizing metabolite concentrations.

## Methods

In the first part of the study patients taking either lithium or valproate were examined using the Cho/Cr ratio method, and both Bipolar Type I and Bipolar Type II patients were included who could also be taking other medications. In the second part of this study only Bipolar Type I patients on valproate monotherapy were included, and quantification of choline concentrations was made. Some of the data from the first part of this study has been reported previously [38].

### Subjects and Study Design

All subjects gave full informed consent, and both studies were approved by the ethics committee at the University of Alberta. Healthy controls were examined using a detailed, but non-standardized, psychiatric interview. They were excluded if there was any personal history, or immediate family history, of psychiatric disorder. For patients, diagnoses were made using DSM-IV criteria for Bipolar Disorder Type I or Type II following detailed psychiatric interview, with additional information being available in almost all cases from long-term psychiatric clinic records. They also had to be taking a dose of either lithium or valproate which maintained their blood levels within the ranges of 0.4–1.2 mmol/l for lithium and 200–700 μmol/l for sodium valproate. Serum lithium and valproate levels were also measured on the day of MRS scanning. Other medications taken by the patient were noted. In the second part of the study the same criteria were used, except that only patients meeting diagnostic criteria for Bipolar Disorder Type I were included, and they had to be on sodium valproate monotherapy. This was done to examine Bipolar Type I patients in more detail, and to remove a possible confounding variable. All patients had to be euthymic for the previous 3 months, as determined by interviews with the patient, and additional interviews with their relatives and bipolar clinic records when available. MRS scans were carried out within 24 hours of this interview.

### Magnetic Resonance Spectroscopy Methodology

For both studies magnetic resonance experiments were performed using a Magnex 3 T scanner with 80 cm bore equipped with actively shielded gradient, and spectrometer control was provided by an Surrey Medical Imaging System (SMIS) console. The subjects head was immobilized with a restraint system. Signal transmission and reception were achieved using a quadrature birdcage resonator for ^1^H measurements.

### Part 1 - Magnetic Resonance Spectroscopy

**Figure 1:**
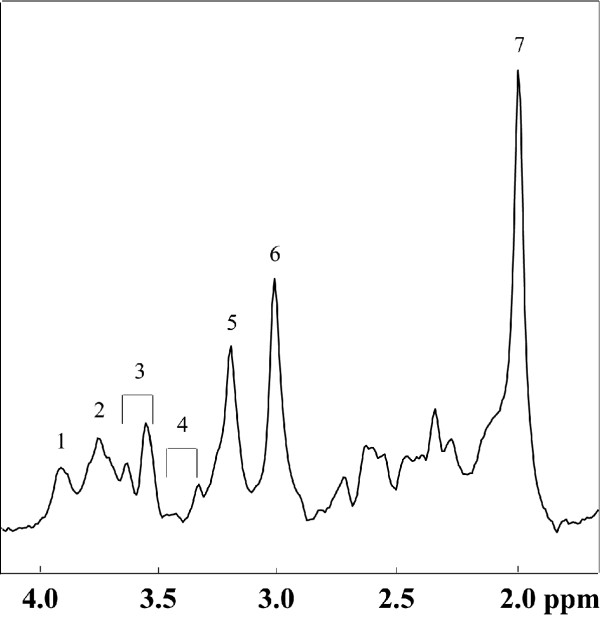
A typical ^1^H-MRS spectrum of the human brain at 3.0 T. A number of metabolites can be seen. 1: creatine (methylene) + phosphocreatine, 2: glutamate + glutamine, 3: *myo*-inositol + glycine, 4: taurine, **5: total choline compounds**, 6: creatine (methyl) + phosphocreatine, 7: N-acetylaspartate.

Initially, MRI data were acquired using gradient echo imaging sequences to produce multiple slice images along both coronal and transverse planes. This allowed registration of a 2 × 2 × 3 cm volume-of-interest (VOI) to be selected in the temporal lobe. ^1^H MR spectra were acquired using the PRESS localization method [39,40], with TE = 32 ms, TR = 3 s, and with 128 averages. Baseline correction and deconvolution of the spectra was accomplished using the Peak Research (PERCH) spectrum analysis software package. The metabolite peaks of interest [choline (Cho) and creatine (Cr)] in each spectrum were fitted to a Gaussian line-shape for peak area estimation. To determine changes in choline concentrations we examined the Cho/Cr ratio. Figure 1 shows an individual ^1^H MRS spectra in which all the major metabolite peaks can be seen.

### Study 2 - Magnetic Resonance Spectroscopy

To accurately quantify the brain concentration of creatine we used a 125 ml glass sphere containing a solution of 4 mmol creatine as an external standard. The PRESS sequence was used to acquire proton MRS data with TE1 = 25 msec, TE2 = 25 msec, TR = 3000 msec, and 128 scan averages. The MRS data were acquired from three 2 × 2 × 2 cm^3 ^voxels placed in the cortex of the left frontal lobe, the cortex of the left temporal lobe, and in the external standard solution. The average coordinates [41,42] of the centers of the two brain voxels were determined: x = 0.5 mm (SD = 1.6), y = 63.5 mm (SD = 12.1), z = -25.5 mm (SD = 4.2) in the frontal lobe, and x= 32.2 mm (SD = 6.3), y = 20.5 mm (SD = 3.9), z = 10.7 mm (SD = 2.6) in the temporal lobe. In order to measure T_1 _and T_2 _values of the metabolites in the brain and external standard solution, MRS data were collected with different TE values at a constant TR and different TR values at a constant TE both for the healthy volunteers and the patients and also from external standard solution [42]. However, due to these constraints, the fact that the two studies used different populations at different times, and the size of the external 125 ml container (which limited voxel size to 2 × 2 × 2 cm^3^), it was not possible to exactly match the voxel size or location between the two studies.

### MRS Data Analysis

For quantitative measurement of brain metabolite concentrations we used previously described methodology [42,43]. In this, [Met]_b_, in millimoles per kg of wet brain, the CSF volume fraction, f_csf_, in the spectroscopic voxels must be corrected. Thus, brain metabolite concentrations were calculated as described in the following equation:



where V_voxel _is the volume of a 8 cm^3 ^spectroscopic voxel [43], and N_b _represents the number of metabolite molecules per unit voxel in brain.

### Statistical Analysis for both MRS studies

Means ± SEM were used in the statistical analysis. Sex differences were analyzed using chi-squared, and age differences with ANOVA with *post-hoc *Tukey tests. The MRS data was analyzed using Student's unpaired *t*-test using a significance level of p < 0.05 comparing diagnostic groups (patients vs controls) in each brain region (frontal and temporal).

## Results

### Study 1

#### Subjects

A total of 18 healthy controls, 14 bipolar patients taking lithium, and 11 bipolar patients taking valproate completed this study. Of the 14 bipolar patients taking lithium, 7 were Type I and 7 were Type II. In the valproate group, 7 were Type I and 4 were Type II. These groups were studied both separately and together, but as there were no statistically significant differences between the Type I and Type II patients, the results for both types are presented together. Of the 14 bipolar patients taking lithium 12 patients were taking other psychotropic medications: these were benzodiazepines (7 patients), antidepressants (5 patients), and antipsychotics (2 patients). Of the 11 patients taking sodium valproate 10 patients were taking other psychotropic medications: these were benzodiazepines (5 patients), antidepressants (5 patients), and antipsychotics (4 patients).

The mean age for the lithium group was 40.43 ± 2.96 years, for the valproate group 35.47 ± 2.27 years, and for the control group was 31.35 ± 2.89 years. These differences were statistically significant (F = 3.68, df = 2, p = <0.05), which was attributable to the lithium group being significantly older than the control group (Tukey *post hoc*, p < 0.05).

There were no gender differences within the groups: 10 females and 8 males in the control group (χ^2 ^= 0.167, df 1, p > 0.05), 5 females and 9 males in the lithium group (χ^2 ^= 1.143, df 1, p > 0.05), and 6 females and 5 males in the valproate group (χ^2 ^= 0.474, df 1, p > 0.05).

Mean serum lithium levels were 0.79 ± 0.06 mmol/l, and the range was 0.46–1.08 mmol/l. The mean serum valproate levels were 508 ± 42 μmol/l, and the range was 210–912 μmol/l.

### MRS Data

#### ^1^H MRS

We utilized the ratio of the choline peak to creatine peak (Cho/Cr) as a primary correlate of Choline concentrations. This result has been reported briefly in a previous publication [38]. The mean Cho/Cr ratio with this measure was 1.46 ± 0.04 for controls, 1.18 ± 0.07 for lithium-treated patients, and 1.12 ± 0.08 for valproate-treated patients. These were statistically significant, with a reduction in ratios occurring in both the control vs. lithium comparison (t = 3.628, df = 30, p = 0.001) and the control vs. valproate comparison (t = 4.248, df = 27, p = 0.002).

### Study 2

#### Subjects

A total of 11 healthy controls and 9 Bipolar Type I patients taking valproate as monotherapy were entered into this study. The mean age for the control group was 37.3 ± 2.2 years, and for the valproate patients 42.4 ± 3.0 years. These differences were not statistically significant (F = 1.49, df = 1, p = 0.27).

There were no gender differences within the groups: 7 females and 2 males in the valproate group and 5 females and 6 males in the control group (χ^2 ^= 0.474, df 1, p > 0.05). The mean serum valproate levels were 472 ± 36 μmol/l, and the range was 284–728 μmol/l.

In the frontal lobe the mean choline concentration for the healthy controls was 2.21 ± 0.17 mmol/kg wet brain and for the patients was 2.38 ± 0.12 mmol/kg wet brain. In the temporal lobe the mean choline concentration for the healthy controls was 2.35 ± 0.14 mmol/kg wet brain and for the patients was 2.40 ± 0.19 mmol/kg wet brain. There were no statistically significant differences between the controls and patients in either the frontal (t = 0.78, df = 18, p = 0.44) or temporal (t = 0.203 df = 18, p = 0.84) lobes (Table 1).

**Table 1:** Concentrations (mmol/kg wet brain) and ratios (Cho/Cre) in frontal and temporal lobes in healthy volunteers and in patients chronically treated with valproate (Study #2)

			Choline (Cho)		Creatine (Cre)		Cho/Cre	
			Frontal	Temporal	Frontal	Temporal	Frontal	Temporal
Healthy Controls	Age	Sex						
1	50	M	3.51	2.95	6.67	8.53	0.53	0.35
2	45	M	2.19	3.03	10.1	9.11	0.22	0.33
3	43	F	3.01	2.31	9.97	9.52	0.30	0.24
4	39	M	2.11	2.72	7.94	7.60	0.27	0.24
5	37	F	2.47	2.34	9.98	9.89	0.25	0.24
6	36	F	1.91	1.76	8.28	8.19	0.23	0.22
7	35	M	1.76	2.36	7.93	8.36	0.22	0.28
8	32	F	1.88	1.51	9.56	9.56	0.2	0.16
9	32	M	1.94	2.14	7.04	7.79	0.28	0.28
10	30	F	1.82	2.52	7.8	8.63	0.23	0.29
11	28	M	1.72	2.23	7.16	8.51	0.24	0.26
**Mean**	**37.00**		**2.21**	**2.35**	**8.40**	**8.70**	**0.27**	**0.26**

Valproate Treated Patients								

1	58	F	2.72	2.1	9.16	10.13	0.30	0.21
2	50	M	2.61	3.42	8.17	10.53	0.32	0.33
3	49	F	2.03	1.79	8.56	7.48	0.24	0.24
4	48	F	2.44	1.88	9.93	8.19	0.25	0.23
5	36	M	2.60	2.53	7.84	7.51	0.33	0.34
6	35	F	2.07	2.77	9.26	10.39	0.22	0.27
7	35	F	2.78	1.89	8.35	9.79	0.33	0.19
8	34	F	1.76	2.93	7.26	8.01	0.24	0.37
9	34	F	2.43	2.27	7.75	7.23	0.31	0.31
**Mean**	**42.11**		**2.38**	**2.40**	**8.48**	**8.81**	**0.28**	**0.28**

The Cho/Cr ratios in the frontal lobes were 0.27 ± 0.028 in controls and 0.28 ± 0.015 in patients. In the temporal lobes the Cho/Cr ratios were 0.26 ± 0.021 in controls and 0.28 ± 0.016 in patients. There were no statistically significant differences between the controls and patients in either the frontal (t = 0.367, df = 18, p = 0.72) or temporal (t = 0.539, df = 18, p = 0.59) lobes (Table 1).

## Discussion

The results from the present study vary considerably between the two sections utilizing differing methodologies. This is despite the fact that both studies were carried out by the same group on the same scanner with bipolar patients coming from the same patient pool. This strongly suggests that the methodology used to determine choline concentrations can considerably alter the results. In the first part of the study we found that both the lithium-treated and valproate-treated patients had significantly reduced Cho/Cr peak ratios compared to controls. This is similar to the findings from one previous study which also suggested that there may be a trend towards decreased choline in grey matter [28]. This study was a frontal lobe study that measured metabolite concentrations in a 1.5 T scanner in bipolar type I patients hospitalized for manic (n = 9) or mixed (n = 8) states. In this study most patients were being treated with valproate and an atypical antipsychotic.

These findings, however, differ from those in the second part of the present study in which we found no differences in choline concentrations between valproate-treated patients and controls in either frontal or temporal lobes. This second part of the study was much better controlled in terms of the patients receiving valproate monotherapy, only including bipolar Type I patients, and in using an external choline solution to accurately quantify choline concentrations. This finding of a lack of change is also in keeping with most previous studies. Several studies which have also previously measured metabolite concentrations with 1.5 T scanners also found no changes. These include a study of the hippocampus in 15 euthymic bipolar type 1 patients, of whom 10 were taking either lithium or valproate [19], a study of basal ganglia in 8 rapid cycling patients on lithium [22], a study of the anterior cingulate in 10 bipolar children [23], and a study in frontal lobes of 23 euthymic bipolar patients of whom 13 were on lithium [25]. Several other studies have examined metabolite ratios, mostly in patients on lithium, and those also found no changes in choline concentrations [20,21,26,27]. In a study using metabolite ratios in bipolar children who were off medication for at least one week there was also no change in choline concentrations [24]. In a double-blind placebo-controlled human volunteer study before and after one week of lithium administration we also found no changes in cholinein 10 volunteers [30], which is similar to a patient study which compared 7 patients on lithium to 6 non-lithium treated controls and in which no differences were seen [29].

In contrast, animal studies have suggested that lithium may increase brain choline concentrations, and in lithium-treated patients it also increases the accumulation of choline within erythrocytes [4-7]. Nonetheless, ^1^H-MRS studies in patients examining this possibility is mixed. To date 6 studies have suggested some support for this [13-18], but in none of these studies were metabolite concentrations measured, and most of the studies measured choline/creatine ratios [14-18], the other one measuring metabolite intensity/tissue volume [13]. The first study to examine brain choline in basal ganglia studied only 4 patients, all of whom were on lithium [18]. Another study examined 19 euthymic inpatients and found increased choline/creatine ratios in basal ganglia, but only 10 of these patients were receiving lithium [17]. The third study to report an increase in this ratio (in this case in the left subcortical region) was in a mixed group of patients receiving a wide range of medications [16]. Two other studies have reported increased choline concentrations, but only in limited circumstances. In one study in 11 bipolar children patients were examined before and after lithium administration [14]. There were no significant findings before or after lithium administration, although there was a trend towards increased choline/creatine ratios in the patients before lithium treatment. This latter finding does not suggest that in patients lithium significantly alters the choline/creatine ratio. The final study examined 15 euthymic males who were on either lithium or valproate [13]. This study found that thalamic choline concentrations, determined by measuring metabolite intensity/tissue volume ratios, were significantly increased only if the right and left hemisphere were compared separately, but not if they were compared together.

It is also conceivable that both lithium and valproate may increase Choline concentrations, but that the differences were not large enough for us to detect, or that without lithium or valproate treatment patients would have lower Choline concentrations. The cross-sectional nature of this study does not allow this to be examined. It is also important to recognize other limitations of the present study. Firstly, these MRS studies are not pre- and post-treatments, so may not accurately reflect changes that occur in individual patients. Secondly, part of the study used a ratio-method to assess choline concentrations, the limitations of which are increasingly clear (particularly since creatine concentrations may be altered by medication [37]). Thirdly, the sizes of all groups are small and it therefore possible that a larger study may have been fully powered to identify differences between groups. Fourthly, several patients in the first study (but not the second study) were on other drugs which may have affected the results of this study. Fifthly, we have not determined if age affects the results, and in the first part the groups were not all matched for age. In addition, the voxel locations were not the same in both studies due to the reasons discussed in the methodology section. Nonetheless, despite these limitations we believe the results add significantly to the literature in this under-researched area.

We conclude that, taking all current evidence together including the findings from the present study, it is unlikely that either lithium or valproate significantly alter brain choline concentrations. However, given the large differences in patients populations, medications received, and MRS methodologies it is difficult to directly compare all these studies. In addition, the methodology used to measure choline concentrations can significantly alter the results. Future MRS studies in bipolar patients should, therefore, examine metabolite concentrations rather than a ratio of choline compared to other metabolites.

## Competing interests

None declared.
